# Clinical Profiles of Dengue Infection during an Outbreak in Northern India

**DOI:** 10.1155/2016/5917934

**Published:** 2016-11-29

**Authors:** Anish Laul, Poonam Laul, Vamsi Merugumala, Ravi Pathak, Urvashi Miglani, Pinkee Saxena

**Affiliations:** ^1^Maulana Azad Medical College, No. 2, Bahadur Shah Zafar Marg, New Delhi, Delhi 110002, India; ^2^Deen Dayal Upadhyay Hospital, Hari Nagar, Clock Tower, New Delhi, Delhi 110064, India

## Abstract

*Introduction.* Dengue fever is an arboviral disease, which is transmitted by mosquito vector and presents as varied clinical spectrum of dengue fever (DF), dengue hemorrhagic fever (DHF), dengue shock syndrome (DSS), and expanded dengue syndrome (EDS) with atypical presentations, thus posing a diagnostic dilemma. Unless we are aware of these presentations, diagnosis as well as early initiation of treatment becomes difficult. We studied the various clinical presentations of dengue infection during an outbreak of disease in 2015.* Materials and Methods*. A total of 115 confirmed cases of dengue infection from Department of Medicine of Deen Dayal Upadhyay Hospital, New Delhi, were enrolled in this observational study.* Results.* The common signs and symptoms of dengue infection were fever, headache, body ache, backache, retro-orbital pain, bleeding manifestations, and rash in 100%, 87%, 86%, 58%, 41%, 21%, and 21%, respectively. Nonspecific or warning signs and symptoms included vomiting, weakness, abdominal pain, breathlessness, vertigo, sweating, and syncope. Other possible signs and symptoms of coinfections, comorbidities, or complications included diarrhea, sore throat, and neurological manifestations. There were seven patients with coinfections and four with comorbidities. The final diagnosis of these patients was DF (73%), DHF (16.5%), DSS (1.7%), and EDS (4.3%). Among EDS patients, the atypical presentations included encephalopathy, lateral rectus nerve palsy, acalculous cholecystitis, and myocarditis. Four patients required ICU care and there was no death in this study.* Conclusion*. Knowledge of atypical presentations is a must for early diagnosis and timely intervention to prevent life-threatening complications.

## 1. Introduction

Dengue is a viral disease of tropical regions. The causative agent is one of serotypes of a single stranded RNA virus called DENV 1, 2, 3, and 4 [1]. It is a tropical disease, which is transmitted by the* Aedes aegypti* mosquito. Incubation period is 4 to 7 days (range 3–14 days) [2]. Once the symptoms start, person can remain infectious for next six to seven days. In addition to mosquito bite, dengue has been reported to be transmitted by transfusion of blood from an infected donor, injuries by infected sharps to health care workers, transplantation of organs and tissues from infected donors, and from infected pregnant mother to her fetus by vertical transmission [3–5]. This viral infection has a wide clinical spectrum ranging from asymptomatic disease to undifferentiated fever (or viral syndromes), classical dengue fever (DF), dengue hemorrhagic fever (DHF), or dengue shock syndrome (DSS) and expanded dengue syndrome (EDS) [2]. After incubation period ends, the disease is followed by three phases: febrile, critical, and recovery phase [2]. In the recent years, dengue has crossed geographic borders and has spread to many new countries. Currently, it is endemic to southeast Asia and western pacific regions [2]. So dengue fever has become a major international public concern particularly in tropical and subtropical regions affecting urban as well as rural areas. Though several measures are taken to prevent and control it, recurrent outbreaks have been reported in India. The first outbreak of dengue fever in India was in 1812 [6].

Several major outbreaks took place after this in years 1836, 1906, 1911, 1972, 2005, 2010, and 2015. An increasing number of cases of dengue are being reported with atypical presentations as frequent epidemics are occurring. As awareness of this disease is increasing, rare manifestations are also being reported. During an outbreak in 2015 in Northern India, the present study was conducted in tertiary care hospital in Delhi highlighting the varied clinical presentations of dengue fever.

## 2. Materials and Methods

This was an observational study conducted on the patients selected from indoor and outdoor Departments of Medicine of Deen Dayal Upadhyay Hospital in West Delhi, India, during the period from 1 June to 31 August 2015. 115 patients suffering from dengue fever during an outbreak of the disease were enrolled. All patients who were admitted with complaint of fever and were found positive for either NS1 antigen (Micro ELISA, J. Mitra) or dengue IgM antibodies (NIV Pune) were included in the present study. A detailed history was taken and careful clinical examination was performed. Besides routine biochemical and hematological investigations [hemoglobin, total leucocyte count (TLC) and differential leucocyte count (DLC), platelet count, hematocrit (HCT), liver function tests (LFT), blood urea, and serum creatinine], malarial antigen, Slide test for malarial parasite, IgM antibodies and Widal test for typhoid, and X-ray chest and ultrasonography (USG) of abdomen were also done in all patients. Other investigations were performed according to the clinical conditions of the patients. All subjects were classified according to WHO guidelines as shown in Table 1 [2].

### 2.1. Criteria for Classification of Dengue Infection

Patients having neurological, cardiac, gastrointestinal, musculoskeletal, renal, ocular, and other nonspecific manifestations were grouped in expanded dengue syndrome (EDS) category. Thrombocytopenia was taken as platelet count less than 1 lac/mm^3^ and leucopenia as wbc ≤5000 cells/mm^3^.

## 3. Results

192 subjects with fever and clinical suspicion of dengue were tested for NS1 antigen and dengue IgM antibody and 115 subjects (59.8%) of confirmed dengue fever with age more than 18 years were included in the study.

## 4. Age-Wise Distribution of Patients

Age distribution of patients is as shown in Table 2. We had 64 (57%) males and 51 (44%) females enrolled in our study.

## 5. Clinical Presentations

As far as clinical spectrum was concerned, all patients had come with fever along with varied clinical manifestations as shown in Table 3. 65 patients (57%) had abdominal pain, out of which 26 had right upper quadrant pain with fever. Nine patients had positive Murphy's sign. All the 26 patients with pain had edematous gall bladder without gallstones. Diagnosis of acalculous cholecystitis was made and all patients responded well to supportive therapy. 29 patients (25%) had associated hepatomegaly and six patients (5%) had ascites associated with hepatosplenomegaly. All 65 patients (57%) of pain abdomen had elevated AST and 49% had elevated ALT as shown in Table 7. There was significant difference in ALT among the four groups with elevated levels indicating the severest and atypical forms of infections in form of DHF, DSS, and EDS (*p* value 0.039) as shown in Table 8. All patients with raised AST and ALT were discharged once they showed falling trend and no patient reported to have any hepatic sequelae.

One patient with jaundice and enzymes in range of 200 IU also recovered completely within two weeks. All other causes of jaundice were ruled out in this. Only one patient in our study had blood urea of 42 mg% and serum creatinine 1.6 mg%; however he also recovered with supportive treatment without any complication. There was no hepatic or renal failure reported in our study.

22 patients (19%) had breathlessness, out of which 18 patients (16%) had pleural effusion on chest X-ray as shown in Table 4. Two patients were known asthmatic, one had come in respiratory failure, and another had dyspnoea due to congestive heart failure.

## 6. Investigations (X-Ray, Ultrasound Abdomen, Test for Coinfections)

For more details see Table 4.

## 7. Blood Investigations

90 patients (78%) had thrombocytopenia with platelet count less than one lac. There was significant difference in the platelet count among the four groups, with DHF and DSS having significantly low values (*p* value 0.003). 80 patients (70%) had leucopenia with relatively decreased lymphocyte count among the four groups, the mean value being the lowest in DSS and DHF group, respectively (*p* value < 0.001) (Tables 5 and 6).

Clinical categorisation of subjects was done as shown in Table 9. Nineteen patients were diagnosed with DHF and two with DSS. 24 patients (20%) had hemorrhagic manifestations in form of epistaxis, hematemesis, hemoptysis, melena, subconjunctival hemorrhage, and menorrhagia. Ten patients required both blood and platelet transfusion and five required only platelet transfusion. A total of thirty units of platelets were transfused. Intravenous fluid therapy was given according to WHO guidelines.

## 8. Clinical Categorisation

Four patients (3%) required ICU care because of DSS, myocarditis, and encephalopathy. One patient of DSS had come with acute respiratory distress in shock with saturation of 79% and needed intubation in casualty and was transferred to ICU and put on ventilator. He had pleural effusion as well as ascites and very low platelet count. He was monitored with central line, transfused 6 units of platelets, and required ICU care for five days. Total hospital stay was eight days and patient recovered well without any residual disease.

Another patient of DSS with BP of 60/40 and haematemesis required ICU care with platelet as well as blood transfusion. His hospital stay was of 6 days. Patient responded well to platelet, blood, and fluid therapy. Another patient of encephalopathy also required ICU care.

The fourth patient requiring ICU care was an eighteen-year-old girl. She had come with fever and breathlessness which on physical examination revealed congestive heart failure. Patient was hypoxemic and hypotensive. She had thrombocytopenia, EKG revealed sinus tachycardia, X-ray chest revealed bilateral pleural effusion, and echocardiography showed pericardial effusion, global hypokinesia, and reduced left ventricular systolic function with ejection fraction of 40%. Clinical diagnosis of myocarditis was made. She was treated in ICU with standard decongestive therapy, positive inotropic support, and platelet infusion. Patient responded well to treatment and had ejection fraction of 56% at the time of discharge with no long term sequelae on follow-up.

Five patients (4.3%) had expanded dengue syndrome (EDS) affecting nervous system (encephalopathy, facial, and lateral rectus nerve palsy) in three, cardiovascular system (myocarditis) in one, and gastrointestinal system in one (jaundice). The patient with encephalopathy was brought to casualty with history of fever and altered sensorium. On examination he was found to be responding only to painful stimuli with normally reacting pupils and fundus examination. His all investigations were normal except low platelet, mildly raised ALT and AST, and positive NS1 antigen. MRI brain was inconclusive. CSF was sterile on culture with lymphocytosis and normal glucose values. Diagnosis of dengue encephalopathy was made. Patient was given supportive care in ICU with strict monitoring of electrolyte and fluid balance. He started to recover after 6 days. Recovery was prolonged with slight slurring of speech which recovered fully within next two months.

One patient reported drooping of saliva and slurred speech and another acute onset of diplopia with squint. MRI was done to exclude intracranial space occupying lesion or bleed. Provisional diagnosis of facial and lateral rectus nerve palsy was made in consultation with ophthalmologist. Both patients responded well to conservative treatment and recovered without any sequelae within eight weeks.

Out of 115 patients, seven had coinfections with malaria in three (confirmed by Slide test positive for* Plasmodium vivax *malarial parasite and malarial antigen) and typhoid in four confirmed with Widal test as shown in Table 3. Four patients had comorbidities with diabetes and asthma (two subjects each). Diabetic patients had their blood glucose monitored regularly. There was no death or long term disability in our study patients.

## 9. Discussion

The present study highlighted the varied spectrum of dengue fever ranging from some known clinical presentations of fever, rash, headache to some atypical presentations like acalculous cholecystitis, encephalopathy, myocarditis, and facial and lateral rectus nerve palsy. The atypical manifestations of dengue fever are increasing in recent outbreaks as evidenced by various studies.

Headache and retro-orbital pain are well-known features of dengue fever. In present study we found that 87% of patients had headache as chief complaint and 41% had typical retro-orbital pain. In study by Mandal et al. [6], 62.16% patients presented with headache. In some studies like Itoda et al. [7], 90% of patients presented with headache. On the other hand, Awasthi et al. [8] conducted a study in north India and reported that only 9% of cases had headache as their chief symptom.

With technological advancements like ultrasonography, more and more cases of dengue fever are being reported with ascites and pleural effusion but it is to be done at right time. We have detected third space collection in form of ascites in 6% and pleural effusion in 16% of patients. In a study by Mandal et al., [6] ascites was present in 8.1% and pleural effusion in 18.9% of cases. In Bangladesh based study by Mia et al. [9], 42% had pleural effusion and 41% of patients developed ascites. In ultrasound based study by Kalayanarooj et al. [10], pleural effusion was diagnosed in 18%, which corroborated with the findings of the present study.

Hemorrhagic manifestations are a well-known complication of dengue fever due to decreased platelet count and leakage from blood vessels. Thrombocytopenia may be because of spontaneous aggregation of platelets to virus-infected endothelium, bone marrow suppression, or immune mediated clearance. In our study we found that 20% of patients had hemorrhagic manifestations in the form of epistaxis, hematemesis, hemoptysis, melena, subconjunctival hemorrhage, hematomas, and menorrhagia. Hemorrhagic manifestations were present in 40% of patients in a study by Karoli et al. [11].

In the present study, 78% of patients had platelet count <1 lakh. Singh et al. [12] observed thrombocytopenia in 61.39% of cases. In a study by Mandal et al. [6], 37.8% had platelet count below 50,000 per cubic mm and 13.51% had hemorrhagic manifestations in the form of melena and gum bleeding. In a study by Tripathi et al. [13], only 12.8% had platelet count <70,000 but 28% cases had hematemesis, 26% had melena, and 14.28% had epistaxis. In a Hyderabad based study by Khan et al. [14], only 5% of patients had bleeding while 40% had thrombocytopenia.

Low leucocyte count may be due to virus-induced inhibition of myeloid progenitor cells or due to destruction. We found that 70% had leucocyte count <5000 almost similar to a study by Itoda et al. [7] in which leucopenia was observed in 71% of cases. Ageep et al. [15] detected leucopenia in 90% of cases while Mandal et al. [6] found leucopenia in 29.73% of cases. Elevated transaminases are found to be raised in a large percentage of our study. Dengue fever can cause hepatic injury and elevation of transaminases. 57% of patients had elevated ALT and 49% had raised AST.

57% of patients presented with pain abdomen along with fever. 22% had acalculous cholecystitis with right upper quadrant abdominal pain, positive Murphy's sign, abnormal liver function tests, and thickened gall bladder wall without stones on ultrasonography. The exact mechanism of acalculous cholecystitis in dengue is not known. It could be due to viral invasion of gall bladder wall causing microangiopathic injury and increased vascular permeability leading to protein rich plasma leakage. This could be the cause of thickening of gall bladder wall [1]. Severity as well as progression of dengue fevers has been found to be significantly associated with thickening of gall bladder wall in some studies. The course of disease is usually self-limiting but, in a few patients, acalculous cholecystitis progresses rapidly to ischemic gangrene and perforation. Cholecystectomy is reserved for such patients who progress to peritonitis.

We had five cases of EDS with neurological, cardiovascular, and gastrointestinal involvement. The unusual atypical manifestations, in this study, were of neurological involvement in 3 (2.6%) patients. Neurological involvement in dengue may occur because of neurotropism of the virus, immunological mechanisms, cerebral anoxia, intracranial hemorrhage, hyponatremia, cerebral edema, hepatic failure, renal failure, or release of toxic products. Peter et al. [16] reported isolated facial nerve palsy in dengue fever and Shivanthan et al. [17] have also reported isolated 6th nerve palsy. Donnio et al. [18] reported dengue with a rare presentation of oculomotor nerve palsy. Sanjay et al. [19] reported a case of optic neuropathy associated with dengue fever.

One patient was categorised as EDS due to myocarditis. This patient had benign though prolonged self-limiting course. Bhasin et al. [20] also reported a case of DHF with myocarditis mimicking DSS with hypotension and pulmonary edema.

One patient reported acute respiratory distress syndrome and needed ICU care. ARDS in dengue is due to increased permeability of alveolar capillary membrane, which results in alveolar edema leading to pulmonary dysfunction [21]. Anam et al. [22] had an unusual EDS case presenting as pancreatitis and haemothorax with respiratory distress. This complication requires early recognition and prompt management for favorable outcome.

## 10. Conclusion

Dengue is a challenging disease with multisystemic, varied, atypical, and sometimes life-threatening presentations. Awareness of these rare manifestations goes a long way in early recognition, correct diagnosis, prompt intervention, and appropriate treatment. Every aspect of dengue viral infection remains a clinical challenge. A continuous seroepidemiological surveillance, timely interventions, vaccines research, and vector control measures are needed to identify the cases so that outbreaks, complications, and mortality can be minimized.

## Figures and Tables

**Table 1:** 

DF/DHF	Grade	Signs and symptoms	Laboratory
DF	With or without haemorrhagic manifestations	Fever with two of the following: (i) Headache. (ii) Retro-orbital pain. (iii) Myalgia. (iv) Arthtralgia/bone pain. (v) Rash. (vi) Haemorrhagic manifestations. (vii) No evidence of plasma leakage.	Leucopenia (wbc ≤5000 cells/mm^3^). (i) Thrombocytopenia (platelet count <150 000 cells/mm^3^). (ii) Rising haematocrit (5%–10%). (iii) No evidence of plasma loss.

DHF	I	Fever and haemorrhagic manifestation (positive tourniquet test) and evidence of plasma leakage.	Thrombocytopenia <100 000 cells/mm^3^; HCT rise ≥20%.

DHF	II	As in Grade I plus spontaneous bleeding.	Thrombocytopenia <100 000 cells/mm^3^; HCT rise ≥20%.

DHF	III	As in Grade I or II plus circulatory failure (weak pulse, narrow pulse pressure (≤20 mmHg), hypotension, restlessness).	Thrombocytopenia <100 000 cells/mm^3^; HCT rise ≥20%.

DHF	IV	As in Grade III plus profound shock with undetectable BP and pulse.	Thrombocytopenia < 100 000 cells/mm^3^; HCT rise ≥20%.

**Table 2:** 

Age groups	Frequency	%
18–20 years	23	20%
21–30 years	48	41.7%
31–40 years	22	19.1%
41–50 years	12	10.4%
>50 years	10	8.7%
Total	115	100%
Mean ± SD	31.36 ± 13.17	
Median	30	
Min–max	13–71	

**Table 3:** 

Symptoms	Number of patients	Percentage%
Fever	115	100
Headache	100	87
Body ache	99	86
Backache	67	58
Retro-orbital pain	47	41
Bleed (any hemorrhagic manifestation)	24	21
Rashes	25	21
Vomiting	78	68
Weakness	79	68
Pain abdomen	65	57
Breathlessness	22	19
Vertigo	16	14
Sweating	15	13
Syncope	14	12
Diarrhoea	31	27
Sore throat	29	25
Neurological manifestations	3	2
Itching	22	19
Others	9	8

**Table 4:** 

Finding	Positive cases	Total	Percentage%
Pleural effusion (PE)	18	115	15
Gall bladder edema (GBE)	26	115	22
Hepatosplenomegaly (HS)	29	115	25
Hepatosplenomegaly + ascites (HS + A)	6	115	5
Malarial antigen (R-MAT)	1	115	0.9
Slide test for malaria	2	115	1.8
Widal test	4	115	3.5

**Table 5:** 

WBC count	Mean ± SD	Median	Min–max
Total	3749.48 ± 2411	3000.00	1100–18500
N	55.58 ± 12.54	55.00	25–90
L	38.83 ± 12.58	40.00	9–64
M	2.78 ± 1.89	2.00	0–10
E	1.95 ± 1.89	1.00	0–16

**Table 6:** 

WBC count	DF		DHF		DSS		EDS		*p* value
	Mean	±SD	Mean	±SD	Mean	±SD	Mean	±SD	
Total	3398.43	±1645.59	4404.21	±2771.25	10550	±11243	4790	±3267.72	0.541
N	53.49	±11.93	63.53	±11.25	64.5	±28.99	59	±11.18	0.011
L	41.49	±12.07	29.42	±9.49	27	±24.04	31.8	±6.8	<0.001
M	2.75	±1.93	3.11	±1.91	3	±1.41	1.75	±0.96	0.499
E	1.99	±2.07	1.74	±1.24	3.5	±0.71	1.5	±0.58	0.265

**Table 7:** 

	Mean ± SD	Median	Min–max
PL count (K)	52.70 ± 38.54	41.00	10–204
HCT	38.86 ± 5.91	39.00	18.10–67.30
UREA	18.97 ± 5.21	18.00	10–42
Creatine	1.16 ± 1.69	1.00	0.20–17
Serum bilirubin	1.13 ± 0.63	1.10	0.20–6.30
AST	69.50 ± 51.52	50.00	24–346
ALT	60.10 ± 38.55	45.00	20–234

**Table 8:** 

	DF		DHF		DSS		EDS		*p* value
	Mean	±SD	Mean	±SD	Mean	±SD	Mean	±SD	
Hb	12.74	±9.81	12.36	±4.16	15.25	±0.35	12.14	±2.14	0.074
PL count (K)	58.24	±41.31	41.11	±15.68	27.5	±17.68	14.4	±3.78	0.003
HCT	38.48	±4.39	39.82	±10.21	43.5	±9.19	40.06	±7.64	0.706
Urea	18.5	±4.75	20.75	±6.22	25	±14.14	19.38	±4.89	0.696
Creatine	1.24	±1.88	0.83	±0.23	0.7	±0.42	0.85	±0.45	0.142
Serum bilirubin	1.18	±0.67	0.89	±0.23	0.7	±0.42	1.08	±0.58	0.042
ALT	62.85	±47.07	89.6	±63.61	128	±11.31	92.2	±65.74	0.039
AST	56.18	±32.61	71.6	±54.61	79	±62.23	80.8	±59.91	0.758

**Table 9:** 

Categorisation	Frequency	%
DF	84	73%
DF with Hg	5	4.3%
DHF	19	16.5%
DSS	2	1.70%
EDS	5	4.3%
Total	115	100%
